# Changes in cerebrospinal fluid interleukin-10 levels display better performance in predicting disease relapse than conventional magnetic resonance imaging in primary central nervous system lymphoma

**DOI:** 10.1186/s12885-020-07774-5

**Published:** 2021-02-22

**Authors:** Yan Zhang, Dongmei Zou, Jingjing Yin, Li Zhang, Xiao Zhang, Wei Wang, Meifen Zhang, Daobin Zhou, Wei Zhang

**Affiliations:** 1 Department of Hematology, Peking Union Medical College Hospital, Chinese Academy of Medical Sciences & Peking Union Medical College, Beijing, China; 2 Department of Hematology, Beijing Hospital, Beijing, China; 3 Department of Clinical Laboratory, Peking Union Medical College Hospital, Chinese Academy of Medical Sciences & Peking Union Medical College, Beijing, China; 4 Department of Ophthalmology, Peking Union Medical College Hospital, Chinese Academy of Medical Sciences & Peking Union Medical College, Beijing, China

**Keywords:** Primary central nerves system lymphoma, Cerebrospinal fluid, Interleukin-10, Monitoring, Relapse

## Abstract

**Backgroud:**

Establishing diagnostic and prognostic biomarkers of primary central nervous system lymphoma (PCNSL) is a challenge. This study evaluated the value of dynamic interleukin (IL)-10 cerebrospinal fluid (CSF) concentrations for prognosis and relapse prediction in PCNSL.

**Methods:**

Consecutive 40 patients newly diagnosed with PCNSL between April 2015 and April 2019 were recruited, and serial CSF specimens were collected by lumbar punctures (LP) or by Ommaya reservoir at diagnosis, treatment, and follow-up phase.

**Results:**

We confirmed that an elevated IL-10 cutoff value of 8.2 pg/mL for the diagnosis value of PCNSL showed a sensitivity of 85%. A persistent detectable CSF IL-10 level at the end of treatment was associated with poor progression-free survival (PFS) (836 vs. 481 days, *p* = 0.049). Within a median follow-up of 13.6 (2–55) months, 24 patients relapsed. IL-10 relapse was defined as a positive conversion in patients with undetectable IL-10 or an increased concentration compared to the last test in patients with sustained IL-10. IL-10 relapse was detected a median of 67 days (28–402 days) earlier than disease relapse in 10/16 patients.

**Conclusion:**

This study highlights a new perspective that CSF IL-10 relapse could be a surrogate marker for disease relapse and detected earlier than conventional magnetic resonance imaging (MRI) scan. Further evaluation of IL-10 monitoring in PCNSL follow-up is warranted.

## Background

Primary central nervous system lymphoma (PCNSL) is a rare extranodal subtype of non-Hodgkin lymphoma (NHL) that accounts for 4% of newly diagnosed brain tumors and 4 to 6% of all extra-nodal lymphomas, with an incidence of 0.4–0.5/100,000 per year [1]. The incidence of PCNSL has increased over the past three decades, especially in elderly patients. Stereotactic biopsy guided by magnetic resonance imaging (MRI), the preferred option for diagnosis, can yield positive results in 90% PCNSL and open biopsy procedures are rarely necessary. Cerebrospinal fluid (CSF) examination is a good option for cases with leptomeningeal involvement. Because of the nonspecific neurologic symptoms, early diagnosis of PCNSL is a challenge and the diagnostic process may be protracted for months to years. More biomarkers have emerged to facilitate diagnosis. Interleukin (IL)-10 plays a role in lymphoma development by promoting B-cell proliferation and inhibiting apoptosis [2–4]. IL-10 may play various roles in the development of lymphoma and high levels of serum IL-10 were associated with poor prognosis in DLBCL; moreover, IL-10 and IL-10 receptors were also identified as novel treatment targets [5–9]. In 1997, Whitcup’s group first reported increased CSF IL-10 in two cases of PCNSL [10], suggesting that CSF IL-10 may act as a biomarker of PCNSL. However, the sensitivity and specificity of CSF IL-10 for diagnosing PCNSL varies across studies. In 2013, Rubenstein and colleagues reported that the bivariate elevation of IL-10 and chemokine (C-X-C motif) ligand 13 (CXCL 13) in CSF is highly specific for PCNSL, with a positive predictive value of 95% and a negative predictive value of 88% [11]. Data from Peking Union Medical College Hospital (PUMCH) revealed sensitivity and specificity of 95 and 100%, respectively, for a CSF IL-10/IL-6 ratio cutoff value of 0.72 [12]. In primary vitreoretinal lymphoma (PVRL), a special subtype of PCNSL, the diagnostic value of the anterior chamber fluid IL-10 concentration and IL-10/IL-6 ratio has been well documented [13, 14].

Furthermore, a 2006 study reported that changes in CSF IL-10 concentration correlate with prognosis after standard first-line therapy [15]. Similar results were reported recently for PCNSL. Song et al. demonstrated that elevated CSF IL-10 levels after two cycles of high-dose methotrexate were associated with shorter progression-free survival (PFS) and Nguyen-Them et al. showed a negative impact on PFS of CSF IL-10 level at end-induction [12, 16]. However, these previous studies lacked serial monitoring of the CSF profiles.

The present study evaluated the value of dynamic CSF concentration changes of IL-10 in patients with PCNSL during treatment and follow-up. We focused on the precedence relationship of elevated CSF IL-10 concentration and positive magnetic resonance imaging (MRI) findings in relapse patients with PCNSL and found that CSF IL-10 abnormities could be detected earlier than surveillance MRI scan detected relapse, suggesting CSF IL-10 as a potential predictor of relapse.

## Methods

### Patients and CSF

Consecutive patients with newly diagnosed PCNSL treated at Peking Union Medical College Hospital between April 2015 and April 2019, were recruited. All cases had mature B cell lymphoma confirmed by brain biopsy, vitrectomy, or CSF flow cytometry (FCM). Patients with human immunodeficiency virus (HIV) and secondary CNS involvement from systemic lymphomas were excluded. Patients who died within two cycles of treatment for any reason were excluded. Baseline characteristics, including age, sex, liver and renal functions, histological diagnosis, performance status, and radiation scans were collected from patient medical records. This retrospective study was approved by the Ethical Committee of Peking Union Medical College Hospital and all patients signed informed consent forms before sample collection.

Serial CSF specimens were collected by lumbar punctures (LP) or by Ommaya Reservoir at pretreatment, during the treatment (every 2 cycles), at the end of treatment (after 6 cycles), and at follow-up (every 3 months for 2 years). The characteristics of the CSF were recorded, including white cell counts, biochemical examination findings, cytological examination findings, and levels of inflammation factors including IL-6 and IL-10. CSF IL-10 concentrations were measured by electrochemiluminescence immunoassay as described previously [12]. The detection threshold of IL-10 was 5.0 to 1000.0 pg/mL.

### Statistical analysis

Treatment response was evaluated according to the criteria set by the International Primary CNS Lymphoma Collaborative Group [17]. PFS was calculated from the first day of induction to either progression or death by any cause or the last follow-up, while overall survival (OS) was measured from the first day of induction to death by any cause or the last follow-up. The date of the last follow-up was September 10, 2019. Patients with missing survival data were presumed dead at the time of the last clinic follow-up if they had documented disease progression or had their treatment strategy changed to palliation. For those patients in remission at the last clinic follow-up, OS was censored at the time of the last follow-up. Baseline parameters between groups were compared using the independent t-, Mann–Whitney U, and chi-square tests, as appropriate. Survival curves were plotted using the Kaplan–Meier method and compared using log-rank tests. Univariate and multivariate Cox regression analyses were conducted for the outcomes of PFS and OS, and the associated hazard ratios (HRs) with 95% confidence intervals (CIs) were reported. All statistical analyses were performed using IBM SPSS Statistics for Windows, version 25.0 and GraphPad Prism 7.0, with *p*-values < 0.05 deemed statistically significant.

## Results

### Demographic information and treatments

This study included a total of 40 consecutive treatment-naïve patients with PCNSL. All patients had B-cell lymphoma and 39 patients were diagnosed by brain biopsy or vitrectomy. One patient was confirmed to have a mature B-cell origin by FCM.

Ocular involvement was present in 15 (37.5%) patients, six (15.0%) of whom had exclusive ocular involvement at presentation. The remaining nine (22.5%) patients had synchronous ocular and brain involvement. Only one patient with leptomeningeal subtype was diagnosed by FCM in this study.

The median age was 58 (range, 24–79) years and 18 (45%) patients were male. Twenty-two (55%) and nine (22.5%) patients were categorized as intermediate- and high-risk according to International Extranodal Lymphoma Study Group score (IELSG) [18].

Six patients with PVRL received rituximab plus lenalidomide (R2 regimen) as first-line treatment while the remaining 34 patients received high-dose methotrexate and rituximab-based regimens.

**Fig. 1:**
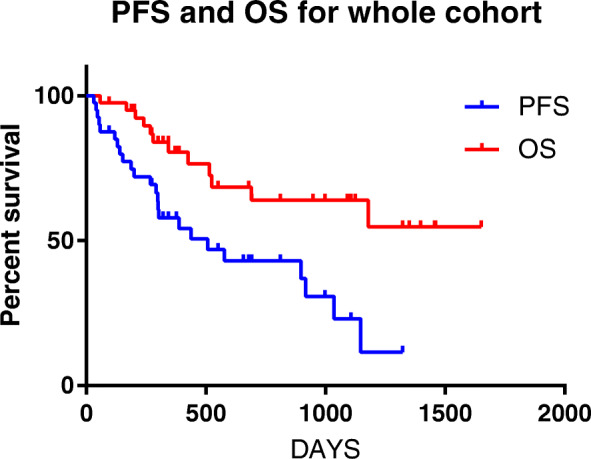
Kaplan–Meier curves for overall survival (OS) and progression-free survival (PFS) of 40 patients

The overall response rate (ORR) of the whole series was 80.0% (32/40), and the complete response (CR) rate was 52.5% (21/40). The median follow-up period was 13.6 (range:2–55) months. At the last follow-up, 24 (60%) patients had relapsed and 12 had died. The median PFS and OS were 617.7 and 1146 days, respectively (Fig. 1).

### CSF IL-10 concentration at pretreatment, interim-induction and end-induction

The first lumbar puncture was performed before treatment in all patients. Eighteen patients had increased intracranial pressure (> 180 mmH_2_) and protein levels were increased in 34/40 patients. Atypical cells were found in 7/40 patients by light microscopy.

The median IL-10 concentration of all patients before treatment was 31.7 pg/mL (range: 5.0–1000) and 34 samples had detectable IL-10 levels (> 5.0. pg/mL). According to our previous report [12], we evaluated the results in this larger cohort and found specificities of 85% (34/40) and 90% (36/40), for a CSF IL-10 concentration cutoff of > 8.2 pg/mL or a CSF IL-10/IL-6 ratio > 0.72, respectively.

**Fig. 2:**
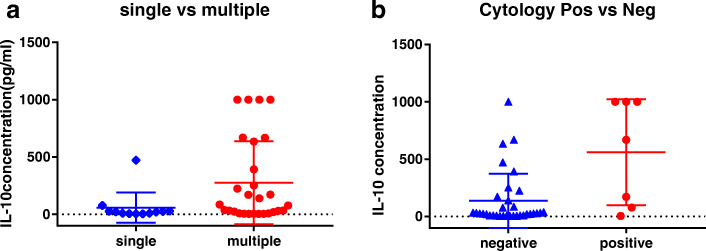
Scatter diagram for the CSF IL-10 concentration between different groups. **a** single lesion group vs. multiple lesions group. **b** CSF cytology negative group vs. CSF cytology positive group

The mean IL-10 concentration was higher in patients with CSF involvement (560.2 ± 174.9 vs. 136.5 ± 41.3 pg/mL, *p* = 0.0113). We observed a higher trend in the multiple-lesion group compared to that in the single-lesion group (275.5 ± 68.42 vs. 59.23 ± 37.96 pg/mL, *p* = 0.053) (Fig. 2) The involved sites (PVRL vs. brain involvement, deep-lesion group vs. superficail-lesion group) and IELSG score (low-, intermediate-, or high-risk) were not related to IL-10 concentration.

The concentrations of CSF IL-10 after two cycles of treatment were tested in 38/40 patients, except for two patients who refused additional LP. The IL-10 concentrations decreased in all but four patients. The mean IL-10 concentration decreased significantly after treatment (220.9 ± 53.62 vs. 67.49 ± 29.1 pg/mL, 95%CI 41.04–265.8, *p* = 0.0088) with a decline of 153.4 pg/mL by paired t-test. The four patients with increased CSF IL-10 levels all relapsed within 6 months (range: 1–6 months).

We observed that a higher IL-10 level after 2 cycles was associated with poor PFS (300 vs. 507 days, p 0.0703), but not with OS (*p* = 0.553).

**Fig. 3:**
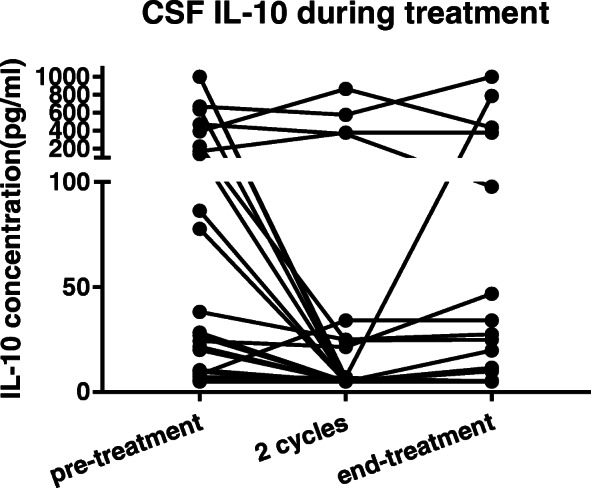
Dynamic CSF IL-10 monitoring during treatment in 29 PCNSL patients. CSF IL-10 mean values during treatment (289.9±358.4 vs. 127.6±203.4 vs 151.1±243.8 pg/bml)

The IL-10 concentrations at the end of induction were measured in 29/40 patients (Fig. 3). Eight patients did not finish induction therapy due to early relapse or severe adverse reactions, and three patients refused LP.

At the end of induction therapy, 21 patients achieved a complete remission (CR), 3 achieved partial remission (PR), 1 patient was stable (SD), and 4 patients had progressive disease (PD). The mean IL-10 concentrations of the CR, PR, and non-response (SD+PD) subgroups were 47.91 ± 37.22, 286.3 ± 120.9, and 219.3 ± 195.2 pg/mL, respectively. There was a trend of lower IL-10 concentration in the CR group compared to that in the non-CR group (47.91 ± 37.22 vs. 244.4 ± 123.8 pg/mL, *p* = 0.0504).

An issue of concern was the relationship between sustained CSF IL-10 concentration and ocular involvement. Six of 21 patients who achieved CR had detectable CSF IL-10 concentrations; all had eye involvement at the time of diagnosis and five had the PVRL subtype. Although the baseline IL-10 concentration was lower in the patients with PVRL, the end-induction of IL-10 concentration was higher in PVRL than that for CNS-involved patients who achieved CR (28.84 ± 17.37 vs. 5.0 pg/mL, *p* = 0.0645).

### CSF IL-10 concentrations increased before disease relapse

Among 24 patients who experienced relapse during the follow-up, 19 had undergone CSF IL-10 measurement. The sensitivity of detectable CSF IL-10 was 78.95% (15/19) in relapsed patients and the mean concentration was lower than the pretreatment level (ΔIL-10: 139.1 ± 295.6 pg/mL, *p* = 0.055, paired t-test).

**Fig. 4:**
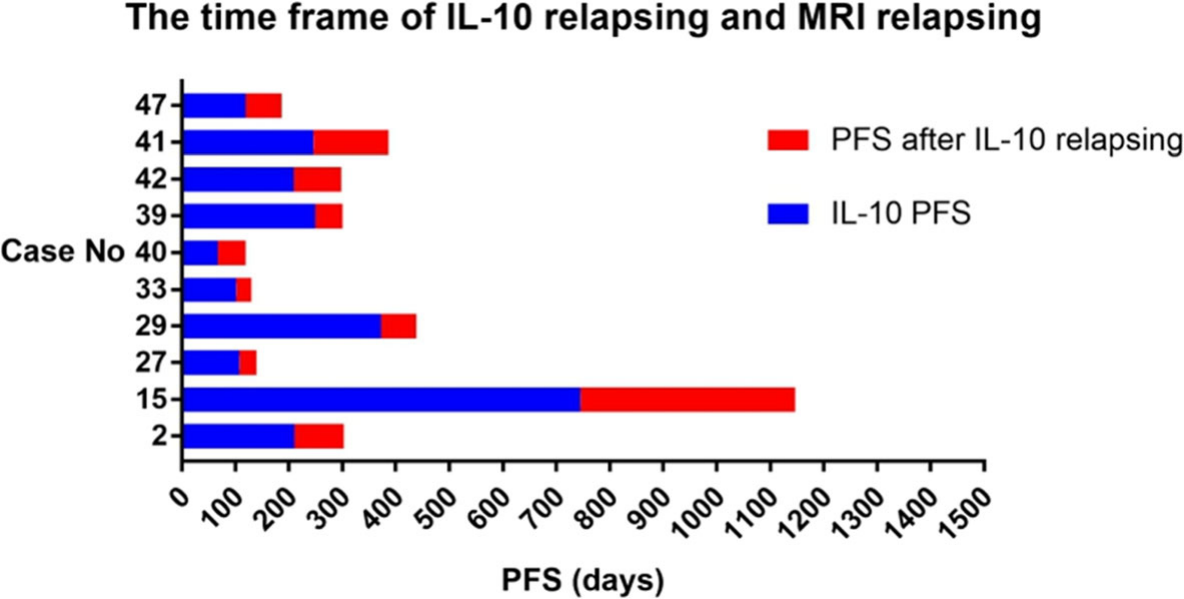
Time Frame of IL-10 relapse and disease progression by MRI scan in 10 patients. Blue: progression-free survival according to IL-10 relapse. Red: the interval after CSF IL-10 relapse and MRI positive findings.

16 patients underwent dynamic CSF surveillance during induction and follow-up. CSF analysis was performed at each induction cycle, which took 3 or 4 weeks, and every 3 months during the follow-up. MRI surveillance was performed every two cycles during the induction phase and every 3 months during the follow-up. Once the patients developed any symptoms that suggested disease recurrence, CSF analyses and MRI scans were performed immediately. We defined IL-10 relapse as a positive conversion in patients with previously undetectable IL-10 and increased concentration compared to the last test in patients with sustained IL-10 levels. As described previously, disease relapse was defined as new CNS lesions on MRI scan and/or new intra-ocular lesions by ophthalmologic examination. IL-10 relapse was detected a median of 67 (range: 28–402) days earlier than disease relapse in 10 patients, while disease relapse occurred at the same time in three patients. Three patients relapsed without detectable IL-10 levels (Fig. 4, Table 1).

**Table 1:** The interval of IL-10 relapse and MRI relapse

Case No.	Lesion location	The time point of IL-10 relapse				PFS by MRI scan(days)	The interval of IL-10 relapse and MRI relapse (days)
		Treatment state	PFS by IL-10 relapse (days)	CSF IL-10 (pg/ml)	Disease status based on MRI		
2	CNS	Follow	210	10.4	PR	303	93
15	CNS	Follow	745	145	PR	1147	402
27	CNS+EYE	Induc	108	27.5	PR	140	32
29	CNS+EYE	Follow	372	28.8	CR	438	66
33	CNS	Induc	102	10.2	CR	130	28
40	CNS	Induc	67	74	PR	119	52
39	CNS	Induc	249	206	PR	300	51
42	PVRL	Follow	209	13.6	CR	298	89
41	PVRL	Follow	246	56.6	CR	387	141
47	PVRL	Induc	119	15.6	CR	187	68

Abbreviations: *CNS* central nerves system, *CR* complete remission, *Follow* follow-up phase, *Induc* induction phase, *PVRL* primary vitreoretinal lymphoma, *PR* partial remission

### Prognostic factors for survival

Cox univariate regression was applied for PFS prognosis analysis using clinical characteristics including ocular involvement, PVRL subtype, IELSG score, treatment regimen, CSF IL-10 levels after two cycles, and post-treatment CSF IL-10 levels. Only detectable CSF IL-10 levels after induction therapy were poor predictors of PFS (*p* = 0.049, HR 0.638, 95% CI 0.408–0.998). The same factors were evaluated by univariate analysis using COX regression for OS. The high-risk group of IELSG showed a trend of poor OS (*p* = 0.077, HR 0.343, 95%CI 0.104–1.124).

## Discussion

The results of this study, based on dynamic monitoring of CSF IL-10 concentrations, are consistent with those of previous studies demonstrating IL-10 as a biomarker of PCNSL. Most importantly, for the first time, we demonstrated that CSF IL-10 changes display better performance in predicting disease relapse than conventional MRI.

Because patients with PCNSL commonly present with nonspecific neurologic symptoms, the diagnostic process may be delayed for months to years. Since 2012, several studies have evaluated the diagnostic value of CSF IL-10 concentration or the ratio of IL-10/IL-6 [11, 12, 19]. Although IL-10 has been identified as a specific biomarker for PCNSL, its origin remains uncertain. Rubenstein et al. demonstrated IL-10 overexpression in lymphoma cells by reverse transcription-polymerase chain reaction (RT-PCR) and immunohistochemistry analysis, while Sasayama confirmed the expression of IL-10 in CD68+ and CD163+ tumor-associated macrophages by double immunostaining analysis [11, 20]. Soussain reported a trend of lower IL-10 levels after corticosteroid treatment [16]. We also observed a rapid decrease CSF IL-10 in several days after corticosteroid administration in our clinical practice; while IL-10 levels may sometimes decrease to the normal range, no change in the tumor is observed on computed tomography (CT) scan. This mismatch between CSF IL-10 decrease and CT scan findings hints that CSF IL-10 is not generated exclusively by tumor cells.

Based on our previous findings, we extended the cohort and performed dynamic surveillance of CSF IL-10 concentrations. Our data are consistent with those of previous studies showing elevated CSF IL-10 concentrations in PCNSL. There is a trend toward decreased sensitivity and specificity of CSF IL-10 when more PCNSL patients were enrolled, and a similar result was observed in our center. Song et al. reported that the diagnostic sensitivity of 95.5% for CSF IL-10 concentration > 8.2 pg/mL in 22 PCNSL patients [12] decreased to 85% when the cohort was expanded to 40 patients. Meanwhile, the positivity rate in relapsed patients 78.9% (19/24), much lower than that in newly diagnosed patients. However, studies differ in IL-10 measurement methods and patient selection; furthermore, many factors can influence CSF IL-10 concentration, including corticosteroid use, disease status, and PCNSL subtype.

We explored new perspectives of CSF IL-10 in the subgroups. Pretreatment IL-10 concentration was associated with tumor burden, as the CSF cytology-positive and multi-lesion subgroups showed higher IL-10 levels. While the prognostic impact on outcome of CSF IL-10 concentration has been widely evaluated in previous studies, the results are controversial. Sasayama et al. reported that high pretreatment IL-10 levels were related to poor PFS, although the cutoff values were different. Nguyen-Them et al. did not observe a negative impact of pretreatment IL-10 level on PFS in a larger cohort and reported that persistent detectable CSF IL-10 at the end of treatment was a negative factor of PFS. The present study obtained results similar to those reported by Nguyen-Them et al. in which only persistent detectable CSF IL-10 at end-induction was associated with poor PFS. The role of prognosis based on CSF IL-10 level requires evaluation in future studies with larger populations, a uniform method, and combined with other potential biomarkers of PCNSL.

The most impressive finding of the present study was that CSF IL-10 relapse was an earlier surrogate biomarker for disease relapsing, a result that, to our knowledge, has not been reported previously. Late relapse is common in PCNSL and more than 25% of patients relapse after 2 years. There remain no accurate factors to predict relapse. Song et al. and Nguyen-Them et al. reported the relationship between increased CSF IL-10 concentration and disease relapse/progression in occasional cases [12, 16]. We performed sequential monitoring by MRI and lumbar puncture in most patients. The data confirmed increased CSF IL-10 concentrations in patients with relapse. We observed that IL-10 relapse occurred a median of 67 days earlier than clinical and/or radiology relapse in 62.5% (10/16) of patients with relapse. This is an encouraging discovery, suggesting that CSF IL-10 is a potential candidate as a predictor of relapse. CSF IL-10 relapse is likely related to micro-lesions in the CNS that cannot be detected by MRI. In 2019, Grommes et al. evaluated the effects of CSF circulating-tumor DNA (ctDNA) and MRI on relapse in surveillance of nine patients with refractory/relapsed CNS lymphomas treated with ibrutinib plus high-dose methotrexate. ctDNA was analyzed using MSK-HemPACT, a custom FDA-authorized next-generation sequencing-based tumor sequencing assay. Only one of nine patients showed ctDNA relapse before MRI relapse and no superiority was found for ctDNA analysis compared to MRI monitoring. CSF IL-10 had a higher sensitivity than conventional MRI scans and novel NGS technology. While CSF IL-10 relapse is not definitive evidence of relapse, this discovery provides a new perspective for PCNSL follow-up. CSF IL-10 concentration can be used as a tool for minimal residual disease. The combination of CSF biomarkers and image surveillance can provide more accurate information on PCNSL relapse. CSF IL-10 monitoring by lumbar puncture is an invasive procedure that limits its use but that can be used to predict disease relapse.

The limitations in this study include the biases inherent to retrospective studies, the limited scale of the cohort, the heterogeneity of therapy regimens, and the lack of follow-up data. The higher proportion of patients with PVRL enrolled in this study may have affected the interpretation of the results. Six (10%) patients had PVRL subtype, five of whom showed sustained CSF IL-10 level at CR. Finally, lumbar puncture is an invasive procedure that is widely used in routine practice.

## Conclusion

In conclusion, this study adds strong evidence to previous studies on the utility of CSF IL-10 levels in PCNSL diagnosis and prognosis. We defined IL-10 relapse and showed the correlation between IL-10 and disease relapse for the first time. CSF IL-10 showed relapse a median of 2-months earlier than MRI showed relapse in most patients, suggesting CSF IL-10 as a potential surrogate biomarker for PCNSL relapse. More research is needed to clarify the origin and impact of IL-10 in PCNSL pathogenesis. The potential role of IL-10 as a surrogate marker for relapse and therapeutic response in PCNSL should also be validated in an independent series along with other potential biomarkers.

## Data Availability

The datasets used and/or analysed during the current study are available from the corresponding author on reasonable request.

## Abbreviations

CSF: Cerebrospinal fluid
CXCL13: Chemokine (C-X-C motif) ligand 13
ctDNA: Circulating-tumor DNA
CR: Complete response
CT: Computed tomography
CI: Confidence interval
DLBCL: Diffused large B cell lymphoma
FCM: Flow cytometry
HIV: Human immunodeficiency virus
HR: Hazard ratio
IL: Interleukin
IELSG: International Extranodal Lymphoma Study Group
MRI: Magnetic resonance imaging
NHL: Non-Hodgkin lymphoma
PUMCH: Peking Union Medical College Hospital
PR: Partial remission
PCNSL: Primary central nervous system lymphoma
PVRL: Primary vitreoretinal lymphoma
PFS: Progression-free survival
PD: Progressive disease
ORR: Overall response rate
OS: Overall survival
